# Supplementary material for: Identification of photoperiod-induced specific miRNAs in the adrenal glands of Sunite sheep (Ovis aries)
Source: Front Vet Sci. 2022 Jul 22;9:888207. doi: 10.3389/fvets.2022.888207 (PMC9354845; doi:10.3389/fvets.2022.888207)
Supplement: Supplementary file 1 [file Data_Sheet_1.ZIP › Supplementary file/miRDeep2 file.zip!/miRDeep2 file/Know_Structure/LP42A1/oar-mir-3959.pdf]

miRBase precursor : oar-mir-3959
Total read count : 15565
oar-miR-3959-5p read count : 13804
oar-miR-3959-3p read count : 1761
remaining reads : 0

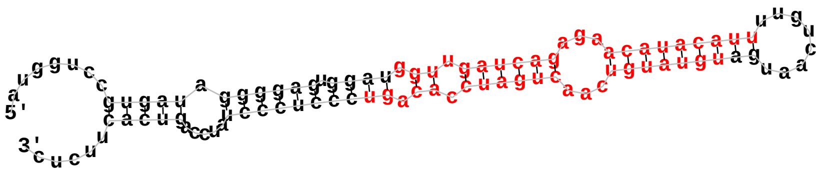

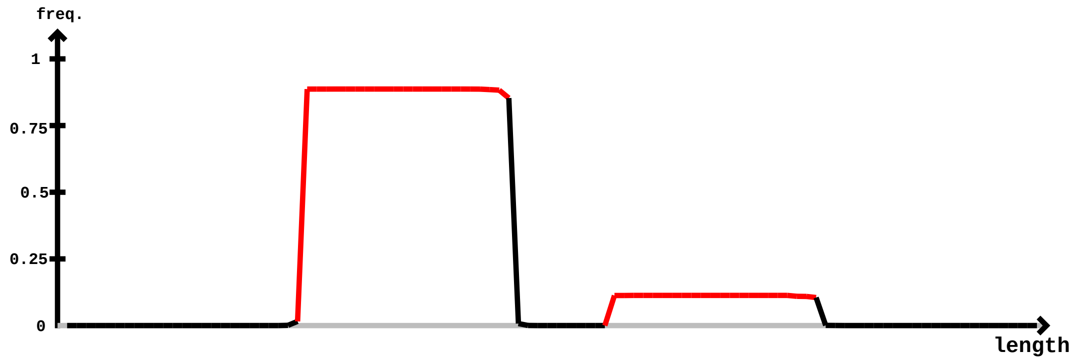


```
                                oar-miR-3959-5p
                                                                oar-miR-3959-3p
5'- augguccgugauaggggaguggauggguugaucagagaacauacauuuugucaaugauguaugucaacugauccacagucccucccuauccagucacuucuc  -3'   exp
    .......(((((.((((((.((((.((.((((((...(((((((((........)))))))))...)))))).)).))))))))))).....))))).....   reads   mm   sample
    ......................augguugaucagagaacauac..........................................................   4       0    seq
    ......................augguugaucagagaacauaca.........................................................   8       0    seq
    ......................augguugaucagagaacauacau........................................................   1       0    seq
    ......................augguugaucagagaacauacauu.......................................................   1       0    seq
    .......................ugguugaucagagaacauaca.........................................................   1       0    seq
    .......................ugguugaucagagaacauacau........................................................   14      0    seq
    .......................ugguugaucagagaacauacauu.......................................................   213     0    seq
    .......................ugguugaucagagaacauacauuu......................................................   3       0    seq
    ........................gguugaucagagaacau............................................................   1       0    seq
    ........................gguugaucagagaacaua...........................................................   3       0    seq
    ........................gguugaucagagaacauac..........................................................   24      0    seq
    ........................gguugaucagagaacauaca.........................................................   23      0    seq
    ........................gguugaucagagaacauacau........................................................   447     0    seq
    ........................gguugaucagagaacauacauu.......................................................   12946   0    seq
    ........................gguugaucagagaacauacauuu......................................................   107     0    seq
    ........................gguugaucagagaacauacauuuu.....................................................   6       0    seq
    .........................guugaucagagaacauacauu.......................................................   1       0    seq
    ...........................ugaucagagaacauacauuuu.....................................................   1       0    seq
    ........................................................uguaugucaacugaucca...........................   1       0    seq
    ........................................................uguaugucaacugauccac..........................   53      0    seq
    ........................................................uguaugucaacugauccaca.........................   7       0    seq
    ........................................................uguaugucaacugauccacag........................   64      0    seq
    ........................................................uguaugucaacugauccacagu.......................   1623    0    seq
    ........................................................uguaugucaacugauccacaguc......................   5       0    seq
    ........................................................uguaugucaacugauccacagucc.....................   1       0    seq
    ..........................................................uaugucaacugauccacagu.......................   2       0    seq
    ..........................................................uaugucaacugauccacaguc......................   1       0    seq
    ..........................................................uaugucaacugauccacagucc.....................   4       0    seq
```
